# Reply to Kulić, Ž. Comment on “Subhadra et al. Significant Broad-Spectrum Antiviral Activity of Bi121 against Different Variants of SARS-CoV-2. *Viruses* 2023, *15*, 1299”

**DOI:** 10.3390/v15112269

**Published:** 2023-11-17

**Authors:** Bobban Subhadra, Ragini Agrawal, Virender Kumar Pal, Agnes-Laurence Chenine, Jeffy George Mattathil, Amit Singh

**Affiliations:** 1 Biom Pharmaceutical Corporation, 2203 Industrial Blvd, Sarasota, FL 34234, USA; 2 Department of Microbiology and Cell Biology, Center for Infectious Disease Research, Indian Institute of Science (IISc), CV Raman Ave., Bengaluru 560012, India; 3 Integrated BioTherapeutics Inc., 4 Research Court, Suite 300, Rockville, MD 20850, USA

We would like to thank Dr. Žarko Kulić [1] for commenting on our publication [2], and we are committed to addressing the points and concerns raised with the utmost diligence and transparency. Our research involving *Pelargonium sidoides* for antiviral bioactive compounds stems from scientific curiosity and the fact that it is known to have broad antiviral activity toward multiple viruses [3,4,5,6]. *P. sidoides*, commonly known as African geranium, is endemic to South Africa [7] and grows along the extreme eastern boundary of the Western Cape, throughout the Eastern Cape, and in parts of the Gauteng, Northwest, Free State, KwaZulu-Natal, and Mpumalanga provinces. Identifying an authentic source of *P. sidoides* was highly important.

We procured multiple lots of *P. sidoides* from Afrigetics Botanicals, a reputed plant botanical production and exporting company in South Africa. The invoice and certificate of analysis of two separate lots of *P. sidoides* are attached (Supplementary Material). For *P. sidoides* extracts used in the study [2], we used the provided protocol for extraction [4] with modifications. We agree with the author’s observation that seasonal and geographical variabilities of plant material can impact the concentration of the compounds, and plant extracts are multicomponent mixtures. Our primary aim was to determine the presence of any antiviral bioactive compounds from *P. sidoides* extracts and identify the compound(s) for future studies and commercialization. We did not claim our *P. sidoides* roots were characterized by chemical fingerprinting. We did not perform any characterization of the plant material used for extraction, as Dr. Kulić mentioned, such as high-performance liquid chromatography (HPLC), thin layer chromatography (TLC), or nuclear magnetic resonance (NMR); however, we did perform HPLC with mass spectrometry to identify the compound.

Regarding the reproducibility of our methods for the *P. sidoides* extracts, we performed the extraction twice. Our initial extraction volume was not large enough due to the higher volume requirement for performing toxicity and neutralization assays; thus, the extraction was repeated twice. We were able to reproduce the extraction methods as described in our manuscript. We agree with Dr. Kulić’s observation that the method for extraction was not thorough; however, we are willing to share with the readers/scientific community a detailed and thorough extraction process. As mentioned above, we were predominantly interested in identifying the compound(s) that showed any antiviral activity in the extracts. Therefore, we initially screened the purified extracts and studied the toxicity and neutralization of SARS-CoV-2 pseudovirus. Based on these results, we studied the neutralization activity of the *P. sidoides* extract using SARS-CoV-2 at IISc Bangalore. We were encouraged by our findings and decided to examine the specific compound more thoroughly to determine if it had a role in the neutralization of SARS-CoV-2. In addition to the *P. sidoides* extraction process, we used reputed research institutions and well-established contract research organizations (CROs) for all the studies/data mentioned in the paper.

We identified the dominant compound in our *P. sidoides* extract as Neoilludin B based on compound library comparison. Neoilludin B was previously reported from *Lampteromyces japonicus* [8]. We did not perform a structural verification of the dominant compound by NMR and our LC–MS data strongly suggest the compound to be Neoilludin B (Figure 1). However, we decided to share our analysis and results with the scientific community because of the wider interest in broad-spectrum antiviral compounds. Based on our data analysis, we ‘putatively’ identified the active compound as Neoilludin B; again, we did not explicitly state that the compound was fully confirmed as Neoilludin B with rigorous structural studies. Plant roots in their natural environment are in constant symbiotic relations with diverse microorganisms, and these interactions between plant roots and the rhizosphere microbiome are critical for plant fitness. New studies have shown that plants and symbiotic bacteria flora produce a plethora of bioactive secondary molecules that play an active role in shaping this two-way metabolic interaction [9,10,11,12]. We are speculating whether any sort of microbial production of Neoilludin B-like molecules in *P. sidoides* root is facilitated by the microbiome. Pelargonium root extracts that we used are not sterile and Bi121 is not produced via sterile processing so the chances of contamination cannot be ruled out in the root and Bi121 preparation; however, the probability of a minuscule amount of contamination-based compound to concentrate and show specific antiviral activity in a targeted assay model is almost nil. Further, regardless of the source of the active compound in the Bi121, we would like to emphasize the fact that we observed a strong antiviral activity of the putatively identified compound towards various strains of SARS-CoV-2, a significant finding that warrants sharing with a wider scientific audience. Our in situ structural studies have provided a scientific basis to corroborate the observed biological activity.

We are currently validating what we observed with more detailed follow-up studies with the Bi121 extract and are also synthesizing the molecular structure to validate the antiviral activity. As part of the scientific community, we agree with the notion that valid scientific studies need to be reproducible. Can other groups reproduce the results that we reported? We have enough *P. sideodes* root powder from which we extracted Bi121 and a sufficient quantity of Bi121 raw extract (Figure 2). We are willing to share this material with any interested research groups under proper material transfer agreements to validate the results. We are currently in the process of synthesizing the compound Neoilludin B to study or corroborate the results we observed. We want to reiterate that we are ready to share our plant material and extracts with anyone interested.

As Dr. Kulić noted in the comment, we acknowledge that the image currently used in Figure 5 in the Subhadra et al., 2023 [2] is not the intended image we had planned to include. We used a figure that was shared with us by the research contract laboratory as a representative image of the HPLC profiles. We sincerely apologize for the inadvertent inclusion of the incorrect image in Figure 5. We deeply regret this oversight. A corrected version of Figure 5 in the original manuscript is included here (Figure 3). While recognizing your concerns, we wish to emphasize our firm commitment to the core findings and study design; our findings have contributed valuable new data regarding the identification of Neoilludin B as a compound in different fractions. Although the HPLC profiles presented in Figure 5 do not impact our study’s results or conclusions, we acknowledge our oversight in using a figure provided by the CRO without sufficient oversight. Once again, we express our sincere appreciation for your engagement with our work, and we are dedicated to addressing all comments and concerns to uphold the highest standards of scientific research.

## Figures and Tables

**Figure 1:**
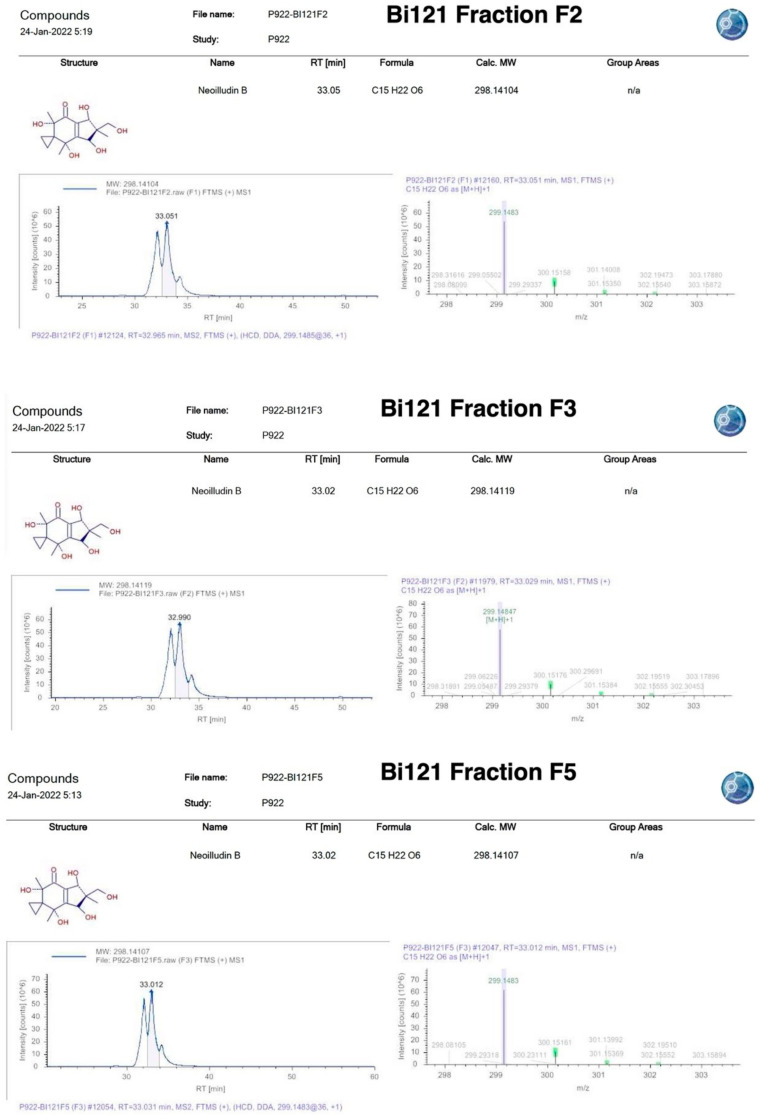
Neoilludin B—LC–MS putative identification from Bi121 fractions (F2, F3, F5).

**Figure 2:**
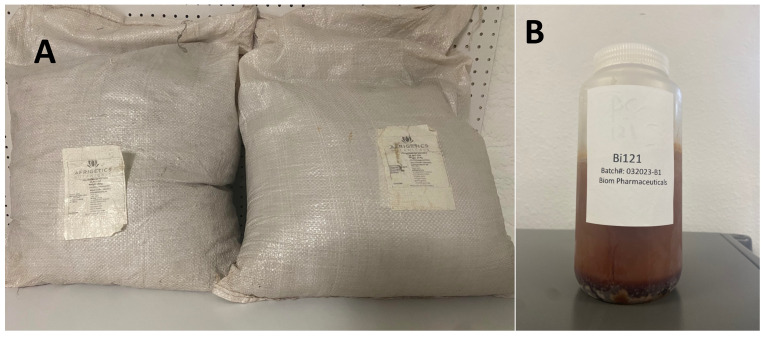
(**A**) *P. sidoides* root powder batch used to prepare Bi121. (**B**) Prepared Bi121 extract from the *Pelargonium* root powder.

**Figure 3:**
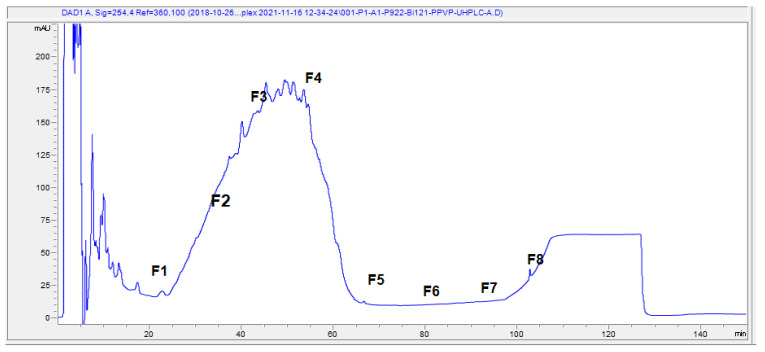
Preparation of polyphenol fractions of Bi121 by reversed-phase UHPLC. Fractionation of the polyphenol-enriched fraction was carried out using an Agilent AdvanceBio Column (2.7 µm, 2.1 × 250 mm) with solvent A (10 mM TEABC, pH 8.0) and an Agilent UHPLC 1290 system. The separation was performed by running a gradient of solvent B (10 mM TEABC, pH 8.0, 90% ACN) and solvent A (10 mM TEABC, pH 8.0) at a flow rate of 250 µL/min. The elute fractions were collected into a 96-well plate using a 1260 series auto-sample fraction collector based on the peaks at UV wavelengths between 214 nm and 280 nm. The 96-well plate elute fractions were collected into 1.5 mL tubes according to retention time (12 min per fraction) for a total of 8 fractions, as shown in the LC chromatography. Six UHPLC runs were performed, and the eight fractions were pooled and further evaporated using a speed vacuum.

## Data Availability

All data and materials are available upon request.

## Supplementary Materials

The following supporting information can be downloaded at: https://www.mdpi.com/article/10.3390/v15112269/s1.
