# Supplementary material for: Reply to Kulić, Ž. Comment on “Subhadra et al. Significant Broad-Spectrum Antiviral Activity of Bi121 against Different Variants of SARS-CoV-2. Viruses 2023, 15, 1299”
Source: Viruses. 2023 Nov 17;15(11):2269. doi: 10.3390/v15112269 (PMC10674820; doi:10.3390/v15112269)
Supplement: Supplementary file 1 [file viruses-15-02269-s001.zip › viruses-2694736-supplementary.pdf]

afrigetics BOTANICALS

PRO FORMA INVOICE

VAT No.: 4560247993

Afrigetics C.C (T.A AFRIGETICS BOTANICALS TM)
381 Waterside Rd
Wilderness
George, Western Cape 6560
South Africa

0448771696
www.afrigetics.com

BILL TO
BULK SALES ENQUIRIES

**Estimate Number:**	Aug 0012
**P.O./S.O. Number:**	DR Bobban
**Estimate Date:**	August 28, 2018
**Expires On:**	September 3, 2018
**Grand Total (USD):**	**$528.50**

Product	Quantity	Price	Amount
**Aloe arborescens** pieces	5	$14.50	$72.50
**Pelargonium sidoides Milled Powder 100ppm**	2	$26.50	$53.00
**Sceletium tortuosum Milled Powder**	2	$135.00	$270.00
**Cites Permit**	1	$60.00	$60.00
**Courier Fees** via EMS	1	$73.00	$73.00

**Total:**	$528.50
**Grand Total (USD):**	**$528.50**

Notes
BANK DETAILS:
Acc Name: Afrigetics cc
Bank: FNB
Branch: George ACC #: 62239920582
Bank Physical Address: 99 York Street, George, 6530.
SWIFT address: FIRNZAJJ

INVOICE DUE BEFORE SHIPPING.

PRO FORMA INVOICE

VAT No.: 4560247993

Afrigetics CC (t/a Afrigetics Botanicals)
22 Waenhout Avenue
Knysna Industrial
Knysna, Western Cape 6570
South Africa

+27 (0) 67 868 8488
www.afrigetics.com

BILL TO
Biom Pharmaceuticals Corporation
Bobban Subhadra
2203 Industrial Blvd,
Saraosta, Florida 34234
United States

505-220-4145
bbobban@gmail.com

Estimate Number: BIOM012022
Estimate Date: January 27, 2022
Expires On: February 11, 2022
Grand Total (USD): **$3,020.00**

Product	Quantity	Price	Amount
**Pelargonium sidoides Milled Powder**	100	$19.50	$1,950.00
**Shipping and handling**<br>Air freight to Florida, USA, CFR incoterms.	1	$1,070.00	$1,070.00

Total: $3,020.00

Grand Total (USD): **$3,020.00**

Notes / Terms
BANK DETAILS:
South Africa:
Bank: First National Bank of South Africa (FNB)
Acc Name: Afrigetics CC
ACC No.: 62239920582 (Branch: George)
Global:
SWIFT address: FIRNZAJJ
FIRSTRAND BANK Ltd, Johannesburg
4 Merchant Place, Corner Fredman Drive and Rivonia
Acc Name: Afrigetics CC
ACC No.: 62239920582
PAYPAL PAYMENTS: bwfqueries@gmail.com

All business is conducted on prepayment unless otherwise agreed.

RAW MATERIAL CERTIFICATE OF ANALYSIS	
**PRODUCT NAME**	PELARGONIUM SIDOIDES MILLED POWDER
**BOTANICAL NAME**	PELARGONIUM SIDOIDES
**FAMILY**	GERANIACEAE
**BATCH NUMBER**	RMPSMP25052021KB
**MANUFACTURING DATE**	MAY 2021
**EXPIRY DATE**	APRIL 2024

PHYSICAL SPECIFICATION	
SENSORY CHARACTERISTICS	Astringent, bitter, woody taste with characteristic Pelargonium tuber smell.
APPEARANCE	Dark brown to reddish free -flowing powder
TEXTURE	Powder
PLANT PART USED	Tuber of the Pelargonium sidoides plant.
COMPOSITION	100% Pelargonium sidoides dried tuber.
METHOD OF PRODUCTION	100% Pelargonium sidoides dried tuber, milled to a powder. Sterilized if required.
IDENTIFICATION	For milled powder:<br>Reduce to a Moderately fine powder. Not less than 95 % by mass passes through a number 355 sieve and not more than 40 % by mass passes through a number 180 sieve and not more than 6 % by mass passes through a number 63 sieve.

ANALYTICAL DATA		
PARAMETER	ACCEPTABLE LEVEL	RESULTS
CHEMICAL CHARACTERISTICS		
Moisture	< 12 %	Complies
Loss on drying	< 12 %	Complies
ASSAY (Umckalin)	> 100 ppm	Complies
Total Ash	< 12 %	11.3%
Ash insoluble in hydrochloric acid	< 3 %	1.74%
Tannins (Expressed as pyrogallol)	>2 %	Complies
**MICROBIAL LIMITS**		
Total Microbial Activity	< 1 000 000 CFU/g	56 500 CFU/g

Salmonella*	Absent in 25g	Absent
Escherichia coli*	Absent in 1g	Absent
Mould	<10,000 CFU/g	350 CFU/g
Yeast	<10,000 CFU/g	150 CFU/g

***** *For where the test result is < 10, it means that no colonies/microbial growth ("No growth") was observed at the lowest/ first dilution (1 in 10 dilution) of the sample. CFU = Colony-Forming Unit (A colony-forming unit is a unit used to estimate the number of viable bacteria or fungal cells in a sample. Viable is defined as the ability to multiply via binary fission under the controlled conditions.)*

HEAVY METALS

Lead	< 5 ppm	Complies
Cadmium	< 1 ppm	Complies
Mercury	< 0.1 ppm	Complies

AFLOTOXINS

Aflotoxins (B_1) are not present.

PESTICIDE RESIDUE

No pesticide residue (Aldrin and Dieldrin) is present.

COUNTRY OF ORIGIN

Product of South Africa

STORAGE, TRANSPORTATION AND STABILITY

STORAGE CONDITIONS	Stored on pallets, labelled, sealed, protected from moisture, light and high temperatures in a pest and rodent-proof area, stored away from harmful chemicals.
TRANSPORTATION	Enclosed vehicle, which is clean, dry, pest and chemical free. Vehicle should be suitable for the transportation of foodstuffs. Vehicle not to present any physical hazards e.g. glass and hard plastics.
SHELF LIFE	36 Months

BULK PACKAGING

MATERIAL	DESCRIPTION
Packaging	In double polyethylene bags placed inside double PVC tamper proof bag.
Net Weight	20 kg

FOOD SAFETY RELATED ACCEPTANCE CRITERIA OR SPECIFICATIONS OF PURCHASED MATERIALS AND INGREDIENTS APPROPRIATE FOR THEIR INTENDED USE

To be used as an ingredient in the preparation of herbal medicine or food supplements.
**STATUTORY AND REGULATORY FOOD SAFETY REQUIREMENTS**
No additional requirements.
**POTENTIAL ABUSE OF PRODUCT**
Storing in direct sunlight, unprotected from moisture and high temperatures. Not tightly closing after opening.

**ALLERGEN STATEMENT**
The above-mentioned product is made of allergen-free ingredients. This product does not contain allergens subject to labelling according to regulation (EU) No. 1169/2011 and amendments. Process controls are in place to ensure that no accidental contamination with allergenic material could occur.
**GMO STATEMENT**
This product has not been in contact with any GMO, therefore the regulations (EC) 1829/2003 and (EC) 1830/2003 are not applicable.
**TSE/BSE STATEMENT**
In the production process of this product, we do not use any raw material of animal origin. This product has not been in contact with and does not include any material of animal origin. Our product is not contaminated with material of animal origin when it leaves our storage facility.
**KOSHER/HALAAL STATUS**
Halaal Certified.

**COMPILED BY**			
**QUALITY MANAGER**	INUS SCHOONRAAD	**SIGNATURE**	**DATE: 25/11/2021**
**MANAGER**	STEVEN HURT	**SIGNATURE**	**DATE: 25/11/2021**
